# An analysis of country adoption and implementation of the 2012 WHO recommendations for intermittent preventive treatment for pregnant women in sub-Saharan Africa

**DOI:** 10.1186/s12936-018-2512-1

**Published:** 2018-10-16

**Authors:** Marianne Henry, Lia Florey, Susan Youll, Julie R. Gutman

**Affiliations:** 1 U.S. President’s Malaria Initiative, U.S. Agency for International Development, Washington, DC USA; 2 Malaria Branch, Center for Global Health, Centers for Disease Control and Prevention, 1600 Clifton Rd. NE, Mailstop A06, Atlanta, GA 30322 USA

**Keywords:** Malaria in pregnancy, Plasmodium, Intermittent preventive treatment, Sulfadoxine–pyrimethamine, sub-Saharan Africa

## Abstract

**Background:**

An estimated 30 million women give birth annually in malaria endemic areas of sub-Saharan Africa. Malaria in pregnancy is associated with an increased risk of adverse maternal and infant outcomes. To combat the adverse effects of MiP, the World Health Organization (WHO) recommends the provision of intermittent preventive treatment in pregnancy with sulfadoxine–pyrimethamine (IPTp–SP) in areas of moderate to high malaria transmission. In 2012, the WHO updated its policy with respect to IPTp administration to recommend administration at each antenatal care visit in the second and third trimesters, with a minimum of three, rather than two, doses. While rapid improvements in coverage were expected, gains have occurred more slowly than anticipated.

**Methods:**

The President’s Malaria Initiative (PMI) assessed IPTp uptake before and after countries implemented the new WHO policy, and assessed how long it took for implementation to occur, using a combination of data from household surveys, routine health management information systems, and programmatic data provided to PMI.

**Results:**

It took an average of 2 years for countries to complete the process of revising their IPTp policies, and it was not until 2015 that all 17 PMI countries had updated their policies. Policy dissemination and training had not been completed in several countries as of early 2018, and only seven countries had fully implemented the new policy including updating their antenatal care registers to collect information on IPTp3+ coverage. The coverage of IPTp1+, 2+, and 3+ has increased by 19, 16, and 13 percentage points since the revised IPTp policy adoption.

**Discussion:**

Overall, coverage of both IPTp2+ and IPTp3+ has improved in recent years. The change in policy from a minimum of two to a minimum of three doses has likely contributed to these improvements. Progress has been slow, likely related to the complicated process of policy adoption exacerbated by the lag in measurement through national household surveys. The impact of future policy changes may be more readily seen if the policy change and implementation process were more streamlined and coordinated between key stakeholders (National Malaria Control Programmes and Reproductive Health Programmes), with more real-time data reporting.

**Electronic supplementary material:**

The online version of this article (10.1186/s12936-018-2512-1) contains supplementary material, which is available to authorized users.

## Background

It has been estimated that 30 million women give birth annually in malaria endemic areas of sub-Saharan Africa [1]. Pregnant women are at increased risk of infection compared to non-pregnant women, and are particularly vulnerable to the adverse effects of malaria infection, which result in an estimated 10,000 maternal and 100,000 newborn deaths annually in sub-Saharan Africa [1, 2]. Malaria in pregnancy (MiP) is associated with an increased risk of severe maternal illness, anaemia, and death, as well as adverse birth outcomes, including premature delivery, miscarriage, stillbirth, low birth weight, and increased neonatal mortality [3].

To address this problem, the World Health Organization (WHO) recommends: (1) distribution of insecticide-treated bed nets (ITNs) to all pregnant women, (2) administration of intermittent preventive treatment in pregnancy (IPTp) with sulfadoxine–pyrimethamine (SP) to HIV negative pregnant women in areas of moderate-to-high endemicity (HIV positive women receive cotrimoxazole for opportunistic infections, which also provides protection against malaria), and (3) provision of prompt diagnosis and effective treatment of malaria and anaemia [4]. The use of ITNs or IPTp in pregnancy reduces the risk of low birth weight by 21% and neonatal mortality by 18% [5].

**Fig. 1:**
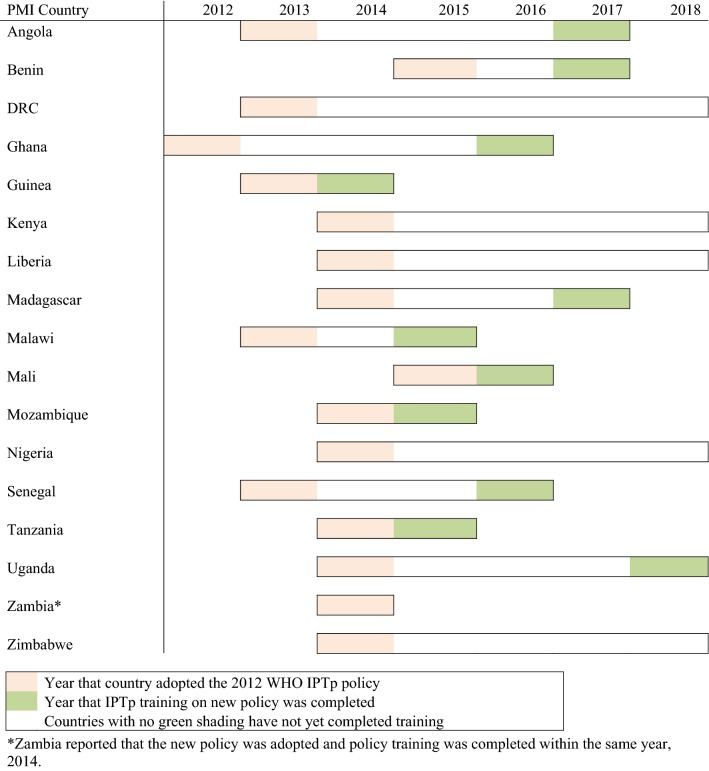
Country IPTp policy adoption and training completion timelines. *Zambia reported that the new policy was adopted and policy training was completed within the same year, 2014

The President’s Malaria Initiative (PMI), a US government initiative launched in 2005 to reduce malaria-related mortality in sub-Saharan Africa, supports National Malaria Control Programmes (NMCP) to implement and scale-up highly effective malaria prevention and treatment measures, including IPTp [6]. From 2010 to 2017, a total of 17 PMI-supported sub-Saharan African countries implemented an updated IPTp policy as part of their strategy for preventing MiP (Fig. 1). IPTp is delivered through an integrated antenatal care (ANC) platform, requiring NMCPs to collaborate closely with reproductive health programmes (RHP). ITNs and IPTp are essential for prevention of MiP. ITNs are ideally provided at the first ANC visit, to ensure access to and use of a net during pregnancy. The WHO recommends that IPTp be provided at each scheduled ANC visit, starting as early as possible in the second trimester, with a minimum of three doses delivered during pregnancy, at least a month apart [7–9]. Prior to 2012, the WHO recommended at least two doses of IPTp, but updated this recommendation in October 2012, based on evidence concluding that provision of three or more doses of SP during pregnancy, rather than two doses, was associated with higher mean birth weight and fewer low birth weight infants [10].

The updated IPTp recommendations were intended to clarify implementation of IPTp, to improve coverage of IPTp, and thus maximize protection during pregnancy. Compliance with the recommendations required countries to update their national guidelines and retrain health providers. In order to track provision of the additional recommended doses of IPTp, updates to ANC registers, HMIS reporting forms, and recording and reporting practices were also required. PMI supported the NMCPs with the policy change and associated processes; by 2015, all 17 PMI countries had updated their national policies (Fig. 1). This paper examines the timing of policy adoption and implementation, as well as trends in IPTp uptake following adoption of the updated policy in 17 PMI countries between 2012 and 2017.

## Methods

The PMI Malaria Operational Plans (https://www.pmi.gov/where-we-work) for the time period FY 2006 to FY 2018 for the 17 PMI countries with an IPTp policy were reviewed to abstract current national IPTp guidelines, timing of new IPTp policy adoption, and status of training. Specifics of each country’s policies were noted, including the recommended timing of the first IPTp dose. In addition, a comparison of the NMCP and RHP IPTp policy was conducted. To assess the roll-out of the updated policy, information was collected regarding the dissemination of the new guidelines to health facilities, the training of health providers, and the process of updating ANC registers and HMIS to include IPTp3+.

The timing of policy adoption and key factors associated with policy implementation were assessed, and changes in IPTp coverage before and after the policy was adopted at the country level were summarized. We assessed four key criteria related to policy adoption and implementation:

1. Official adoption of the 2012 WHO IPTp policy.
2. Dissemination of updated IPTp guidelines to all health facilities.
3. Completion of planned initial health provider training on the updated IPTp guidelines.
4. Collection of IPTp3+ by routine health reporting systems.

National statistics on ANC attendance and IPTp coverage derived from household surveys [demographic and health surveys (DHS), malaria indicator surveys (MIS), and multiple indicator cluster surveys (MICS)] and routine health management information systems (HMIS) were compared for periods before and after adoption of the 2012 WHO IPTp policy. Operational indicators derived from national household surveys were as follows:

- IPTp1+, 2+, and 3+: the proportion of women 15–49 years who had a live birth in the 2 years preceding the survey who received at least one, two, or three doses of SP, respectively, for prevention of malaria during the most recent pregnancy;
- ANC1+, 2+, 4+: the percentage of women who had a live birth in either the two (for MICS) or 3 years (for DHS and MIS) preceding the survey who had at least one, two, or four antenatal care visits, respectively;
- Mean gestational age at first ANC attendance: the self-reported mean gestational age at first ANC attendance, among all women attending any ANC visit.

The baseline survey was defined as the most recent survey available before the country adopted the IPTp policy, including the year it was adopted. The endline survey includes the most recent survey available at least 1 year after the country officially adopted the IPTp policy. Countries that did not have both a baseline and endline survey within these timeframes including data on number of IPTp doses were excluded. Benin, DRC, and Mali did not have post-policy change surveys, thus, 14 of the 17 PMI countries were included in the IPTp coverage analysis for before and after policy adoption. Averages across countries were calculated as the mean of the country coverages.

As national surveys are conducted infrequently, and the ANC and IPTp data collected are retrospective, an additional analysis was done using HMIS data. In most countries, health facilities send monthly reports to the district level including information on numbers of ANC consultations and IPTp doses distributed. The information is collated first at the district level and subsequently at the national level. As the proportion of health facilities reporting data to HMIS has increased substantially in many countries over the study period, analysis was restricted to countries and years in which over 80% of expected health facility reports were received by the HMIS (Additional file 1). Using these criteria, 10 of the 17 PMI countries were included in the HMIS analysis (Angola, Ghana, Kenya, Liberia, Madagascar, Malawi, Mozambique, Senegal, Tanzania, and Uganda). Data on the number of antenatal care visits and IPTp doses administered were used to calculate estimates of IPTp2+ coverage from 2012 to 2016. The denominator for estimates of IPTp coverage from HMIS data varies by country, but is generally defined as the number of women who attended a first ANC visit in a given month. Thus, the operational indicator for IPTp2+ derived from HMIS data is the number of pregnant women attending ANC at a health facility who received two or more doses of SP for prevention of malaria, divided by either the number of pregnant women attending a first ANC visit or the estimated number of pregnant women residing in the facility catchment area.

## Results

### Policy adoption and implementation

#### Official country adoption of the 2012 WHO IPTp policy

In 2012, when the WHO released its updated IPTp policy recommendation, 15 countries had IPTp policies promoting at least two doses; only two countries—Ghana and Zambia—had national IPTp policies promoting at least three doses. In 2013, six PMI countries officially adopted an updated IPTp policy in line with the new WHO guidelines. By 2014, 15 countries had updated IPTp policies, and by 2015, all 17 PMI countries had updated policies (Fig. 1). On average, it took 2 years to complete the process of adopting the 2012 WHO IPTp policy recommendation.

A total of ten countries followed WHO guidance with regard to recommending initiation of IPTp starting at 13 weeks or as early as possible in the second trimester, while the other seven recommended initiation at 16 weeks or during the second trimester (Table 1). Only ten countries have harmonized IPTp guidance across the malaria and reproductive health programmes. RHP guidelines in the remaining countries were not aligned, suggesting limited coordination between the NMCP and the RHP.

**Table 1:** Recommended timing of IPTp initiation and harmonization between NMCP and RHP

Country	Recommended timing of initiation of IPTp per NMCP guidance	Harmonization between NMCP and RHP guidance
Angola	13 weeks	Yes
Benin	16 weeks	Yes
DRC	After first trimester	No; RHP guidance pending IPTp update
Ghana	Second trimester	No; RHP says 14 weeks
Guinea	13 weeks	Yes
Kenya	13–16 weeks	No; RHP promotes 16 weeks
Liberia	13 weeks	Yes
Madagascar	Early as possible in second trimester	No; RHP guidance pending IPTp update
Malawi	16 weeks	Yes
Mali	13 weeks	No; RHP guidance pending IPTp update
Mozambique	13 weeks	Yes
Nigeria	Early as possible after first trimester	No; RHP does not mention IPTp
Senegal	Second trimester	Yes
Tanzania	After 12th week of gestational age	Yes
Uganda	Second trimester	Yes
Zambia	16 weeks	Yes
Zimbabwe	Beginning of second trimester	No; RHP says first dose should be given after quickening

*RHP* reproductive health programme

#### Dissemination of updated IPTp guidelines to all health facilities

Just over half (11, or 65%) of the countries have disseminated the updated IPTp guidelines to health facilities. Of the remaining PMI countries, four have partially disseminated IPTp guidelines (Angola, Madagascar, Malawi, and Uganda) and two plan to begin within the next year (DRC and Zimbabwe) (Table 2).

**Table 2:** Progress in policy adoption and implementation processes by country

Country	1. Official country adoption of the 2012 WHO IPTp policy	2. Dissemination of updated IPTp guidelines to health facilities	3. Completed planned health provider training on IPTp policy	4. Routine health reporting system updated to collect IPTp3
Angola	✓		✓	✓
Benin	✓	✓	✓	✓
DRC	✓			✓
Ghana	✓	✓	✓	✓
Guinea	✓	✓	✓	✓
Kenya	✓	✓		
Liberia	✓	✓		
Madagascar	✓		✓	✓
Malawi	✓		✓	
Mali	✓	✓	✓	✓
Mozambique	✓	✓	✓	
Nigeria	✓	✓		
Senegal	✓	✓	✓	✓
Tanzania	✓	✓	✓	✓
Uganda	✓		✓	
Zambia	✓	✓	✓	✓
Zimbabwe	✓			

#### Completion of health provider training

As of 2017, 12 PMI countries had completed training health providers on the new IPTp policy. An additional three countries projected completing this training within the next 2 years, while Nigeria reported training as ongoing (Fig. 1). Zambia completed both the policy adoption process as well as training of all health providers within the same year. In addition to training health providers in the public sector, five countries—Benin, Guinea, Madagascar, Nigeria and Uganda—also trained private sector providers as part of their national policy implementation process.

#### Updated routine health reporting system to collect IPTp3

**Fig. 2:**
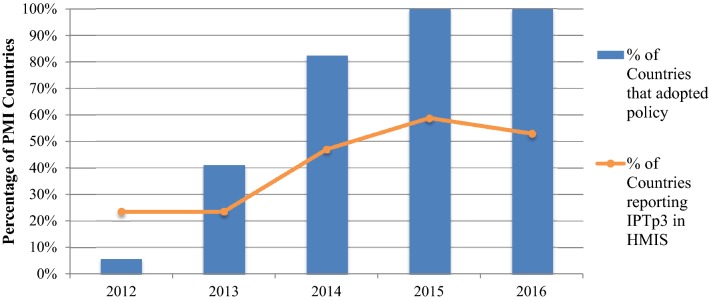
2012 WHO IPTp policy adoption and IPTp3 reporting

Although all 17 countries had adopted the new IPTp policy by 2016, only nine (53%) countries had updated their HMIS to capture the number of women receiving IPTp3+ at health facilities (Fig. 2). In 2012, in accordance with the original monitoring recommendation from the WHO, most countries reported on uptake of at least the first and second doses of IPTp. Ghana and Zambia, countries with consistently high IPTp coverage, have been capturing the number of women receiving the third dose of IPTp since 2008. Between 2012 and 2016, the number of countries reporting uptake of IPTp3+ increased from four to nine. By 2017, all but three countries (Nigeria, Uganda and Zimbabwe) had updated ANC registers to include the third dose of IPTp (Fig. 2). However, without revisions to monthly HMIS reporting forms, facility-level data on IPTp3+ may not be reported up to the national level [11], as is the case in five countries (Benin, Kenya, Liberia, Malawi, and Mozambique) (Table 2). In the context of changing IPTp policies and rapid roll-out and strengthening of national HMIS, IPTp coverage estimates are not standardized. This complicates the use of HMIS data for tracking IPTp coverage at the global level. For example, Mozambique reported the number of women receiving 1, 2 and 3 doses of IPTp until 2015, but in 2016, the HMIS reporting forms were updated to report only 2 and 4 doses. In 2015, Madagascar added reporting of IPTp3 to the HMIS; however, in 2016 they stopped reporting the number of women receiving the first and second dose of IPTp and only retained the third dose.

#### Summary of country policy adoption and implementation processes

Since the new WHO IPTp recommendations were released in 2012, all 17 PMI countries have completed the policy adoption process, and most have begun the process of policy implementation. However, only seven countries—Benin, Ghana, Guinea, Mali, Senegal, Tanzania, and Zambia—have completed all four key criteria of policy adoption and implementation (Table 2).

#### IPTp coverage and ANC attendance before and after policy adoption

**Fig. 3:**
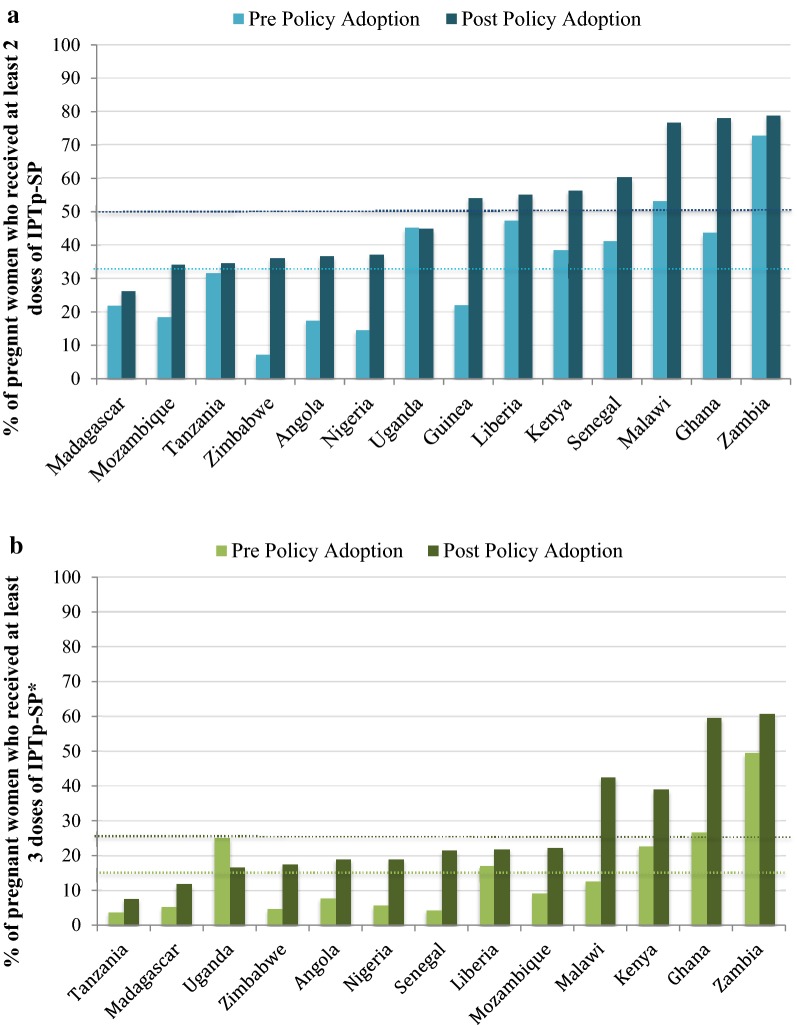
IPTp2+ (**a**) and IPTp3+ (**b**) coverage pre and post country policy adoption in 14 PMI countries as measured by national household surveys. Dotted lines represent the average IPTp coverage across all countries pre- and post- policy adoption; 34% and 50% for IPTp2+, and 15% and 28% for IPTp3+, respectively. Pre Policy Adoption: most recent survey available before or on the year of policy adoption (2008–2014). Post Policy Adoption: most recent survey available that is at least 1 year after policy adoption (all are within 4 years after policy adoption; 2015–2017). Note that the time interval between the pre- and post-surveys varied between countries. *There were no data on IPTp3 coverage for Guinea, thus IPTp3 data represent only 13 countries

Adoption and implementation of the updated WHO policy is temporally associated with improved uptake of IPTp as measured by cross sectional household surveys. Among the 14 countries with appropriately timed baseline and endline surveys, the proportion of women protected by at least one, two, and three doses of IPTp has increased by 19, 16, and 13 percentage points since IPTp policy adoption (Additional file 2; Fig. 3).

The change in IPTp2+ and IPTp3+ coverage from baseline to endline varied across countries (Fig. 3), with coverage of IPTp2+ increasing from a range of 0 in Uganda to 34 percentage points in Ghana, and coverage of IPTp3+ varying from a decrease of 8 percentage points in Uganda to an increase of 33 percentage points in Ghana.

**Fig. 4:**
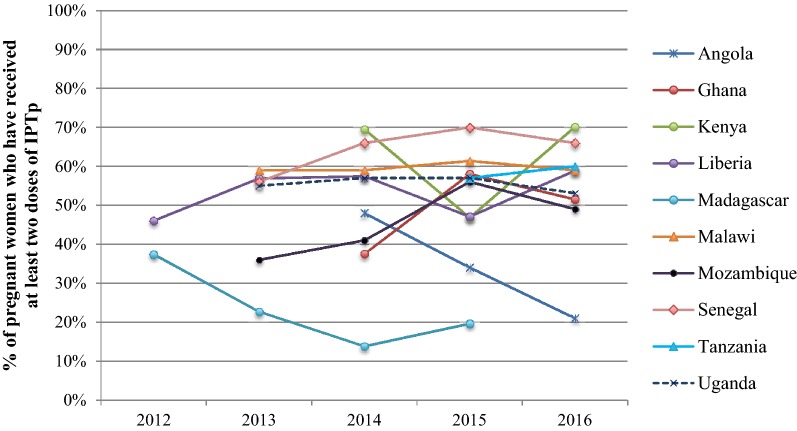
IPTp2+ coverage from 2012–2016 as measured by HMIS in selected PMI Countries with at least 80% reporting completeness

Overall, HMIS data do not show any trend of increased IPTp2+ coverage from 2012 to 2016 in the ten countries with greater than 80% reporting rates (Fig. 4). Since 2012, three countries report decreased coverage (Angola, Madagascar, and Uganda), and one country reported no change in IPTp2+ coverage (Malawi). In a few cases, dramatic fluctuations in IPTp2+ coverage rates are evident. IPTp2+ coverage in Kenya and Liberia decreased drastically from 2014 to 2015, but recovered in 2016. Clear increases in IPTp2+ coverage were only noted in four of the ten countries: Senegal, Mozambique, Ghana and Tanzania.

Overall, there was little change in ANC attendance rates from before to after policy change. ANC attendance is high for one and two visits, but drops substantially for four visits: 93%, 89%, and 59% in the pre-policy change period and 89%, 86%, and 60% in the post-policy change period for at least 1, 2, or 4 ANC visits, respectively (p > 0.05 for all) (Additional file 2). Mean gestational age at the time of first ANC visit dropped slightly, and non-significantly, from 4.7 to 4.4 months (just over a week earlier; p = 0.46). Initiation of ANC prior to 5 months gestational age was associated with higher coverage of IPTp than initiation at or after 5 months (IPTp2 coverage 48% vs 28%, p = 0.03; IPTp3 coverage 22% vs 10%, p = 0.12).

## Discussion

This paper outlines the process for rolling out new global policies across countries and highlights the complexities that affect the pace at which any expected change in outcomes is likely to be observable. While a few countries quickly adopted new IPTp policies in 2012 or 2013, the majority of countries updated their policies in 2014 and 2015. Furthermore, it took more than 2 years, on average, from policy adoption to implementation; in some countries, implementation was much slower, and is still ongoing in four countries. Even in Malawi, one of the earliest adopters of the updated policy, the process to develop and implement the new policy was prolonged and complex [12]. Rapid increases in IPTp coverage following changes in WHO recommendations are not a realistic expectation given the length of time required for countries to make the necessary administrative and logistical adjustments.

In order for a policy change to have maximal impact, the expectations for health care workers should be very clear [13]. A 2013 review, conducted prior to IPTp policy change in five countries, highlighted that there were substantial discrepancies in the guidance put forth by NMCP and RHP, and called for better collaboration and harmonization between these divisions [14]. It was hoped that as the new guidance was rolled out, countries would take the opportunity to ensure harmonization between NMCP and RHP; however, this has not transpired, as just over half of the PMI-supported countries either reported discrepant policies between the two divisions or were unable to provide the RHP policy.

Monitoring the progress of IPTp implementation requires that health care workers are accurately recording SP doses administered in ANC registers and that these numbers are appropriately aggregated and reported monthly from the facilities to the district health offices. Although the recent WHO Malaria Surveillance, Monitoring and Evaluation guidance recommends that IPTp3+ be reported by routine systems, as of this review, only half of the countries assessed are currently reporting IPTp3+ [15].

Finally, the importance of ANC as a platform for this intervention cannot be underestimated. In order for women to receive three doses of IPTp, they must attend at least three ANC visits, ideally beginning as early as possible in the second trimester, with earlier initiation of ANC associated with higher IPTp coverage. While the proportion of pregnant women attending at least one ANC visit is extremely high across the countries assessed, less than two-thirds of women attend four visits, and the majority of women initiate ANC well into the second trimester. Furthermore, IPTp is frequently not administered even when women do attend ANC [16]. Low access to and use of ANC services have been shown to contribute to the poor uptake of IPTp [17]. WHO recently released new guidance on improved pregnancy care advocating for a total of eight contacts during pregnancy, a substantial increase from the previously recommended four visits [18]. Implementation of this guidance will hopefully increase ANC attendance as well as IPTp coverage; however, clear guidance to health care workers will be required as it will not be appropriate to give IPTp at each visit when visits are occurring at intervals of less than a month.

While the scale-up of IPTp coverage has been slow over the last decade, the updated WHO IPTp policy recommendations appear to have contributed to renewed efforts and measured progress in recent years. All 17 PMI focus countries implementing IPTp have updated their policies, though only seven have fully implemented the policy change, according to the four key criteria. National household survey data from the 14 PMI countries with both pre- and post-adoption surveys show a modest increase in IPTp coverage. While this improvement cannot be directly attributed to the policy change, the improvements in overall coverage mirror the timing of the policy change, suggesting that the policy change was likely one of the factors contributing to these improvements. Further, countries with longer standing policies, such as Malawi, and those who moved earlier to a policy advocating three doses, such as Zambia and Ghana, have higher coverage of IPTp.

Trends in IPTp coverage using HMIS data over this period of policy change were not as clear. Only four of the ten countries included in the analysis reported increased IPTp2+ coverage between 2012 and 2016. In Kenya and Liberia, significant fluctuations in IPTp2+ coverage were revealed. One explanation for decreased or fluctuating coverage is stock-outs of SP. For example, in Kenya, widespread stock outs of SP from July 2014 until September 2015 likely led to a decrease in coverage of IPTp, which improved in 2016 once SP was again available. Similarly, in Madagascar, there were central level stock-outs for the majority of 2015–2017, contributing to the decline in IPTp2+ coverage. These trends are evident in the HMIS data as it is a more current data source compared to household surveys.

On-going issues with the availability of SP for IPTp at the ANC clinic and health facility levels may also have contributed to the slower than expected progress on IPTp uptake and coverage. While SP stock outs at the central level are markedly less frequent (on average, 2–3 of the 17 PMI IPTp countries report central level stock outs quarterly), SP stock outs at peripheral level persist. Therefore, supply chain and logistic management information systems need to be coordinated with the policy adoption and implementation processes to ensure sufficient SP is available for administering IPTp to pregnant women.

### Limitations

Policy adoption and implementation are complex processes that are difficult to measure and categorize. The timelines created for this analysis are based on the best available information, which varied considerably between countries. Specifically, the year training began and data on training progress (e.g. percentage of health workers trained in a specified area) were often not available. Similar information gaps were also identified for other steps along the policy implementation process: updating and disseminating guidelines, and rolling out updated reporting systems.

Analysis of IPTp coverage was limited by available data sources: national household surveys and routine information systems. National household surveys have the advantage of being representative of the entire population; however, recall bias likely leads to underestimates of current IPTp coverage, as data from the preceding 2 years are used to calculate coverage estimates. Routine health data may provide an incomplete representation of the population as it is limited to those who attend health facilities. This is exacerbated if private clinic attendance is high as representation of private facilities is incomplete in most countries’ HMIS. Furthermore, the range in IPTp reporting standards (i.e., longitudinal vs daily registers, which are compiled differently), and in denominators used for the underlying populations (i.e., women actually attending first ANC in a given month vs estimated number of pregnant women living in a health facility catchment area), complicate comparisons of HMIS data with national household survey data or with data from other countries. Ideally, the IPTp denominator should exclude ineligible pregnant women, for example, HIV+ pregnant women taking cotrimoxazole, from the denominator, but few countries have made this adjustment to IPTp coverage estimates. This is also not addressed in estimates of IPTp coverage from household surveys. Depending on the overall prevalence of HIV among women of reproductive age in a country, this may have minimal to substantial implications for measuring valid coverage estimates of IPTp.

## Conclusion

Overall, coverage of both IPTp2+ and IPTp3+ has improved in recent years. The 2012 WHO policy promoting delivery of IPTp at each ANC visit (starting in the second trimester) with a minimum of three doses has likely contributed to the improvements in uptake. However, progress has been slower than expected, likely related to the complicated process of policy implementation combined with the lag in measurement through national household surveys and incompleteness of the policy implementation process (e.g. revisions to ANC registers, completing training of health workers). Countries can use these findings to specifically assess and target interventions to those health systems components (capacity building, training, supervision) where progress has been slow and where coverage indicators lag behind. The impact from future policy changes may be more readily seen if the policy change and implementation process could be more streamlined and coordinated between key stakeholders (NMCP and RHP), ensuring real-time data reporting and timely roll-out of training followed by routine supervision. The process of adopting and implementing a complex new policy takes considerable time and effort by all stakeholders and should be factored into any policy change decision. Expectations for observing changes in uptake as a result of a policy change should take these timelines into account. Lessons learned from this process will be considered as the same PMI countries undertake a similar process of adopting new ANC Guidance into their national guidelines and strategies.

## Additional files

**Additional file 1.** Reporting Completeness: Proportion of expected monthly facility reports received by HMIS (%).

**Additional file 2.** Year of policy adoption, training status, and IPTp and ANC coverage for the 13 countries with both pre- and post-policy change surveys.
