# Supplementary material for: An analysis of country adoption and implementation of the 2012 WHO recommendations for intermittent preventive treatment for pregnant women in sub-Saharan Africa
Source: Malar J. 2018 Oct 16;17:364. doi: 10.1186/s12936-018-2512-1 (PMC6192297; doi:10.1186/s12936-018-2512-1)
Supplement: Supplementary file 1 — Additional file 1. Reporting Completeness: Proportion of expected monthly facility reports received by HMIS (%). [file 12936_2018_2512_MOESM1_ESM.docx]

**Additional file 1: Reporting Completeness: Proportion of expected monthly facility reports received by HMIS (%)**

Values in **bold** highlight years when percentage of reporting completeness was greater than 80%

| Country | 2012 | 2013 | 2014 | 2015 | 2016 |
| --- | --- | --- | --- | --- | --- |
| Angola | N/A | 0.76 | **0.82** | 0.76 | **0.82** |
| Ghana | 0.53 | 0.76 | **0.85** | **0.94** | **0.93** |
| Kenya | **0.81** | **0.84** | **0.88** | **0.91** | **0.92** |
| Liberia | **0.80** | **0.83** | 0.73 | **0.80** | **0.92** |
| Madagascar | **0.81** | **0.85** | **0.82** | **0.83** | **0.91** |
| Malawi | 0.72 | **0.93** | **0.97** | **0.97** | **0.91** |
| Mozambique | 0.70 | **0.88** | **0.91** | **0.94** | **0.98** |
| Senegal | **0.93** | **0.94** | **0.94** | **0.97** | **0.99** |
| Tanzania | 0.20 | 0.42 | 0.76 | **0.90** | **0.94** |
| Uganda | 0.69 | **0.91** | **0.97** | **0.99** | **0.97** |

Source: Table 12 "Routine Surveillance Indicators" in PMI Malaria Operational Plans for Fiscal Years 2013-2017
