# Supplementary material for: An analysis of country adoption and implementation of the 2012 WHO recommendations for intermittent preventive treatment for pregnant women in sub-Saharan Africa
Source: Malar J. 2018 Oct 16;17:364. doi: 10.1186/s12936-018-2512-1 (PMC6192297; doi:10.1186/s12936-018-2512-1)
Supplement: Supplementary file 2 — Additional file 2. Year of policy adoption, training status, and IPTp and ANC coverage for the 13 countries with both pre- and post-policy change surveys. [file 12936_2018_2512_MOESM2_ESM.docx]

**Additional file 2: Year of policy adoption, training status, and IPTp and ANC coverage for the 13 countries with both pre- and post-policy change surveys**

| Country | Year Policy Adopted | Training completed | Pre-Policy change survey | Post-policy change survey | Pre-policy adoption | | | | | | | Post-policy adoption | | | | | | |
| --- | --- | --- | --- | --- | --- | --- | --- | --- | --- | --- | --- | --- | --- | --- | --- | --- | --- | --- |
|  |  |  |  |  | IPTP coverage (%) | | | ANC coverage (%) | | | GA @ 1st ANC | IPTP coverage (%) | | | ANC coverage (%) | | | GA @ 1st ANC |
|  |  |  |  |  | 1+ | 2+ | 3+ | 1+ | 2+ | 4+ |  | 1+ | 2+ | 3+ | 1+ | 2+ | 4+ |  |
| Angola | 2013 | 2017 | 2011 MIS | 2015-16 DHS | 28.3 | 17.5 | 7.9 |  |  |  |  | 53.9 | 36.8 | 19 | 80.4 | 76.9 | 60.2 | 4.1 |
| Ghana^**^ | 2012 | 2016 | 2011 MICS | 2016 MIS | 56 | 43.7 | 26.8 | 96.7 | 95.1 | 86.6 |  | 85.3 | 78 | 59.6 |  |  |  |  |
| Guinea^**,%^ | 2013 | 2014 | 2012 DHS | 2016 MICS | 30.8 | 22 |  | 86.9 | 82.5 | 56.3 | 4.2 | 65.2 | 54.1 |  | 74.7 | 70.1 | 50.6 |  |
| Kenya^*^ | 2014 | Ongoing | 2014 DHS | 2015 MIS | 61.9 | 38.6 | 22.9 | 95.7 | 92.1 | 55.8 | 5.4 | 76.6 | 56.4 | 39.1 | 92.7 | 89.3 | 61.5 |  |
| Liberia | 2015 | Ongoing / 2017 | 2013 DHS | 2016 MIS | 64.8 | 47.6 | 17.1 | 94.1 | 92.3 | 77.6 | 3.3 | 82 | 55 | 22 | 95.1 | 92.4 | 77.1 | 3.1 |
| Madagascar^*^ | 2014 | 2017 | 2011 MIS | 2016 MIS | 34.1 | 21.9 | 5.4 |  |  |  |  | 44.7 | 26.3 | 11.9 |  |  |  |  |
| Malawi | 2013 | 2015 | 2012 MIS | 2017 MIS | 76.2 | 53.2 | 12.7 |  |  |  |  | 91.8 | 76.7 | 42.6 |  |  |  |  |
| Mozambique | 2014 | 2015 | 2011 DHS | 2014-15 MIS | 34.9 | 18.6 | 9.2 | 90 | 85.2 | 49.5 | 5.5 | 51.4 | 34.2 | 22.4 | 85 | 81.3 | 53.8 |  |
| Nigeria | 2014 | Incomplete | 2013 DHS | 2015 MIS | 22.6 | 14.6 | 5.8 | 63.6 | 61.7 | 51 | 5 | 46.6 | 37.2 | 19 |  |  |  |  |
| Senegal | 2013 | 2016 | 2012-13 DHS | 2016 DHS | 73.5 | 41.3 | 4.5 | 94 | 88.3 | 45.8 | 3.7 | 87.5 | 60.3 | 21.5 | 95.3 | 90.8 | 52.5 | 3.6 |
| Tanzania^#^ | 2014 | 2015 | 2011-12 AIS^#^ | 2015-16 DHS | 59.6 | 31.8 | 3.9 | 97.7 | 94.1 | 42.8 | 5.4 | 68.3 | 34.6 | 7.7 | 97.5 | 93.4 | 49.2 | 5.5 |
| Uganda | 2014 | Incomplete | 2014-15 MIS | 2016 DHS | 59.3 | 45.2 | 25.2 |  |  |  |  | 76.9 | 45 | 16.8 | 97.7 | 95.1 | 60.2 | 4.7 |
| Zambia | 2014 | 2014 | 2013-14 DHS | 2015 MIS | 88.4 | 72.7 | 49.6 | 97.7 | 95.7 | 54.1 | 4.8 | 90.1 | 78.8 | 60.8 |  |  |  |  |
| Zimbabwe^*^ | 2014 | Incomplete | 2010 MIS | 2016 MIS | 13.3 | 7.3 | 4.8 |  |  |  |  | 46.2 | 36.2 | 17.6 |  |  |  |  |

^#^Tanzania pre-policy change ANC numbers come from the 2010 DHS

*Kenya, Madagascar, and Zimbabwe have subnational IPTp programs; reported coverages reflect results from areas implementing IPTp.

**Guinea 2016 MICS and Ghana 2011 MICS ANC coverage estimates represent 2 years of preceding the survey whereas the other surveys represent 3 years preceding the survey.

^%^Guinea- the drop in ANC attendance from 2012 to 2016 likely reflects the impact of the Ebola epidemic (2013-2015)
